# Another Life Elixir.—French Scientist's Alleged Discovery of Secret of Youth

**Published:** 1900-03

**Authors:** 


ARTICLE II.

### ANOTHER LIFE ELIXIR.—FRENCH SCIENTIST'S ALLEGED DISCOVERY OF SECRET OF YOUTH.

The Paris correspondent of the Morning Post describes a remarkable discovery which has just been made at the Pasteur Institute.

He says that Professor Metchnikoff is engaged in seeking accurate doses of a series of lymphs, each of which will rejuvenate a particular organ of the human body. The Professor objects to premature publicity and insists that the correspondent only say that he had hope. The correspondent says:

"Professor Metchnikoff's experiments show that the explanations of senile atrophy has hitherto been erroneous. The theory was that certain blood cells devoured others and the vital functions began to weaken. The organic poisons thrown off energetically in youth were believed to remain in the system in old age, or at least to be less energetically ejected. These poisoned the finer cells, while without action on those of the conjunctive tissues. The noble cells died, became the prey of the other or plebian cells, thus bringing atrophy to the organ where the metamorphosis occurred. Professor Metchnikoff has proved conclusively that the noble cells are not dead in the organs atrophied by senility.

"Moreover, they may be multiplied. If assisted in their struggle with the plebian cells, they continue to live actively, as in youth, and, theoretically, the organism will cease to grow old and life will be prolonged. Professor Metchnikoff has found means of affording this resistence, and the results already obtained are extraordinary.

"The discovery was made in the following manner:

M. Bordet, one of the Professor's pupils in 1898, published the results of a curious experiment, which consisted of injecting the blood of a rabbit into a guinea pig. Later he injected the blood of this guinea pig in a rabbit and the latter died. Professor Metchnikoff sought the causes of the phenomenon, and was soon convinced that the blood of the guinea pig, injected into a rabbit or other vertebrate animal, elaborates the poison that weakens the red globules of the blood and makes them the prey of the phagocytes.

"Starting from the fact that the poison elaborated in the guinea pig is fatal in large doses, Professor Metchnikoff argued that the action in small doses must be stimulating. On this is based the action of all medicines, such as strychnine and arsenic.

"He therefore, began to inject into rabbits feeble solutions of previously injected guinea pig's blood. A cubic millimeter of the blood of the rabbits thus treated contained before the injections 3,000,000 red globules. In three or four days the number increased to 8,000,000.

"A sovereign remedy against anæmia has been discovered and the theory concerning the globules has been confirmed. An entire section of the Pasteur Institute is now working to find the specific serums for each particular organ. If the blood serum acts on the red globules of the liver the serum must have a similar effect on the cells of the liver, and this is likewise true of other organs. The experiments have demonstrated this.

"A specific kidney serum was found some days ago. The Professor is now determining the exact dose for medical purposes. The discovery has now passed the period of mere laboratory experiments. The celebrated Dr. Vidal is now at work on human serums."

The correspondent draws attention to the obvious result, the great prolongation of human life, if everything is successful.

